# Establishment of a rapid and highly sensitive direct-RAA-RDB detection platform: application in non-deletion α-thalassemia

**DOI:** 10.3389/fmolb.2026.1840485

**Published:** 2026-05-29

**Authors:** Yan Wei, Yilian Zhao, Chao Ye, Mengru Xie, Xiaoxing Zhou, Jinghui Ma, Xingchu Liu, Jilin Qing, Zhizhong Chen

**Affiliations:** 1 Department of Laboratory Medicine, Fengjie Hospital Affiliated to Chongqing Three Gorges Medical College and People’s Hospital of Fengjie, Chongqing, China; 2 Joint Inspection Center of Precision Medicine, The People’s Hospital of Guangxi Zhuang Autonomous Region and Guangxi Academy of Medical Sciences, Nanning, Guangxi, China; 3 Department of Clinical Laboratory, The First Affiliated Hospital of Guangxi Medical University, Key Laboratory of Clinical Laboratory Medicine of Guangxi Department of Education, Nanning, Guangxi, China; 4 School of Clinical Medicine, Guilin Medical University, Guilin, Guangxi, China; 5 Center for Reproductive Medicine and Genetics, The People’s Hospital of Guangxi Zhuang Autonomous Region and Guangxi Academy of Medical Sciences, Nanning, Guangxi, China

**Keywords:** direct-RAA, gene detection, NaOH, RDB, thalassemia, whole blood

## Abstract

**Introduction:**

Recombinase-Aided Amplification (RAA) is widely applied in genetic diagnostics owing to its simplicity and speed, making it a primary choice for Point-of-Care Testing (POCT). However, standard protocols require nucleic acid extraction. By incorporating Reverse Dot Blot (RDB) technology, multiple loci can be detected in a single tube.

**Methods:**

Our team developed Direct-RAA-RDB, a specific and sensitive gene detection platform that eliminates the need for nucleic acid extraction. The applicability of the platform was evaluated for the detection of non-deletion α-thalassemia, focusing on three genotypes: HBA2: c.369C>G (α^WS^α), HBA2: c.427T>C (α^CS^α), and HBA2: c.377T>C (α^QS^α).

**Results:**

The results showed that the Direct- RAA-RDB assay possesses strong specificity and high sensitivity while remaining simple to operate. The entire process is completed within 143 min, allowing for the rapid detection of non-deletion α-thalassemia in clinical settings. Because the platform does not require extraction reagents, extractors, or thermal cyclers, it can be performed in a constant-temperature water bath shaker.

**Discussion:**

This method significantly reduces equipment and reagent costs and offers broad potential for diverse genetic detection applications.

## 1 Introduction

Molecular diagnostic techniques are now standard in disease diagnosis, with PCR and qPCR being the most established methods given their high accuracy and sensitivity (Guo et al., 2020; Guo et al., 2023). However, these techniques require sophisticated instrumentation and dedicated laboratory environments. The rising demand for immediate detection has driven the rapid development of isothermal amplification techniques, such as Recombinase Polymerase Amplification (RPA), loop-mediated isothermal amplification (LAMP), Recombinase-Aided Amplification (RAA), and Rolling Circle Amplification, which offer operational simplicity, speed, minimal equipment requirements, and high sensitivity (Sun et al., 2024). RAA is based on the principle of recombinase-mediated isothermal amplification, which involves the use of specific primers to accurately amplify nucleic acid sequences (Fan et al., 2020). Recombinases can bind to and complex with primer DNA. As primers locate matching sequences on the template DNA, single-stranded DNA-binding proteins (SSB) assist in unfolding the double-stranded structure. In the presence of DNA polymerase, a new complementary DNA strand is synthesized, resulting in exponential amplification (Zhao et al., 2023; Gao et al., 2025). In RAA, DNA or RNA can be amplified within 5–30 min at isothermal temperatures between 37 °C and 42 °C (Li et al., 2024). Instead of a sophisticated PCR instrument, this process requires only a constant-temperature water bath shaker or a dry bath incubator. This transition does not significantly compromise sensitivity or specificity, thereby serving as an efficient and accessible method for Point-of-Care Testing (POCT).

Nucleic acid extraction is a standard requirement during the sample preparation stage for both PCR and RAA. This process involves specific extraction reagents and instrumentation, often necessitating repeated adsorption and elution steps. The establishment of a POCT method that eliminates these steps would reduce cross-contamination between specimens (Custer and Dibner, 2020), simplify operations, and shorten overall detection times. We envisioned that performing some simple pretreatment of samples would allow the processed material to be used directly in the amplification reaction. This approach bypasses the time-consuming DNA extraction phase, reduces the costs associated with specialized reagents and instruments, and minimizes the risk of sample cross-contamination inherent in traditional extraction processes.

The Reverse Dot Blot (RDB) hybridization technique involves the use of molecular hybridization to identify specific loci within amplified products through dedicated probes. These products carry a biotin marker that covalently binds to streptavidin-labeled horseradish peroxidase (HRP). This complex catalyzes the oxidation of tetramethylbenzidine (TMB), producing a visible color reaction for visualization of the results (Hu et al., 2023). Because one streptavidin molecule can bind multiple biotin molecules with extremely high affinity, the system can effectively amplify the detection signals. RDB allows for the immobilization of multiple gene probes on a single membrane, enabling the simultaneous detection of multiple loci in a single tube (Zhang et al., 2021). This capability permits the differentiation between simple heterozygotes and compound heterozygotes with point mutations. Thus, RDB provides high-throughput data by detecting multiple target sequences during a single hybridization reaction.

Therefore, we propose the establishment of a rapid and high-throughput platform for the direct detection of gene mutations in whole blood without DNA extraction. We have designated this system, which combines RAA with RDB technology, as the Direct-RAA-RDB platform. Although we recently reported a similar system for β-thalassemia (Ye et al., 2025), its application for non-deletion α-thalassemia has not yet been described. In this study, we extend the platform to the simultaneous detection of three common non-deletion α-thalassemia point mutations. In the experimental procedure, samples were used directly for RAA amplification after simple pretreatment with NaOH. The entire process removes the need for expensive instruments such as nucleic acid extraction systems and PCR thermocyclers, which are replaced by a constant-temperature water bath shaker or a hybridization chamber. Using non-deletion α-thalassemia as an example, the targeted mutation loci included three genotypes: HBA2: c.369C>G (Hb Westmead; α^WS^α), HBA2: c.427T>C (Hb Constant Spring; α^CS^α), and HBA2: c.377T>C (Hb Quong Sze; α^QS^α). We believe that this platform is applicable for improving testing timeliness and expanding the diagnostic scenarios for thalassemia.

## 2 Methods

### 2.1 Sample collection and extraction

Clinical samples were obtained from the People’s Hospital of Guangxi Zhuang Autonomous Region. The samples consisted of dot-blot images whole blood collected in EDTA anticoagulation tubes on the same day as clinical use. The study protocol was approved by the Ethics Committee of the People’s Hospital of Guangxi Zhuang Autonomous Region (Approval No.: KY-KJT-2023-110). Genotypes were determined using a thalassemia gene test kit (Shenzhen Yaneng Bio-Technology Co., Ltd). Whole-blood genomic DNA was extracted using a DNA extraction kit (TIANGEN), and DNA concentration and purity were determined using a NanoDrop 2000 spectrophotometer (Thermo Fisher Scientific). The extracted DNA was stored at −20 °C for subsequent analysis.

### 2.2 Preparation of standards

Human α-globin gene sequences were obtained from the National Center for Biotechnology Information (NCBI; https://www.ncbi.nlm.nih.gov/). Sequences containing the three mutant genotypes (α^WS^α, α^CS^α, and α^QS^α) were synthesized and cloned into the pUC57 vector (Sangon Biotech Co., Ltd), incorporating an ampicillin resistance gene for selection. The constructed plasmids were transformed into competent cells using the heat shock method. Positive clones were screened on LB agar medium supplemented with ampicillin. Following overnight culture, the bacteria were harvested by centrifugation for plasmid extraction. The accuracy of the amplified plasmids was confirmed by sequencing (Sangon Biotech Co., Ltd).

### 2.3 RAA primer and probe design

RAA primers and probes were designed using SnapGene software based on the human α-globin gene sequence obtained from NCBI. A pair of primers was designed to amplify products encompassing the α^WS^α, α^CS^α, and α^QS^α mutation sites. Given the high sequence homology between the α1 and α2 globin genes, the primers were specifically engineered to differentiate between them and accurately amplify the α2 globin gene fragment. Probes were designed to differentiate each genotype based on the point mutations in the α^WS^α, α^CS^α, and α^QS^α variants. For convenience, the wild type is referred to as “N” and the mutant type as “M.” The target SNPs were identified using six different probes covering three distinct regions of the target gene. All primers and probes were synthesized by Sangon Biotech Co., Ltd. Both forward and reverse primers were labeled with biotin at the 5′ end, while the 5′ end of each probe was labeled with an amino group (–NH_2_). The sequences of the primers and probes used in this study are detailed in Table 1.

**TABLE 1:** Primer and probe sequences.

Item	Name	Sequence (5′–3′)
Primer	A2F	GTGACCCTGGCCGCCCACCTCCCCGCCGAGTTCA
	A2R	TTATTCAAAGACCAGGAAGGGCCGGTGCAAG
Probe	WSN	GGGAGGCGTGCACCGCA
	QSN	GAACTTGTCCAGGGAGGC
	CSN	GGCTCCAGCTTAACGGTATTT
	WSM	GGAGGCCTGCACCGCAG
	QSM	GCCTCCCCGGACAAGTTC
	CSM	CCAAATACCGTCAAGCTGGA
	AC	CTCGGTAGCCGTTCCTCCTG
	NC	ACACCAACCGCATCGTCAT
	CC	CACATCACACACTCTGCGAC

### 2.4 Sample pretreatment

**FIGURE 1:**
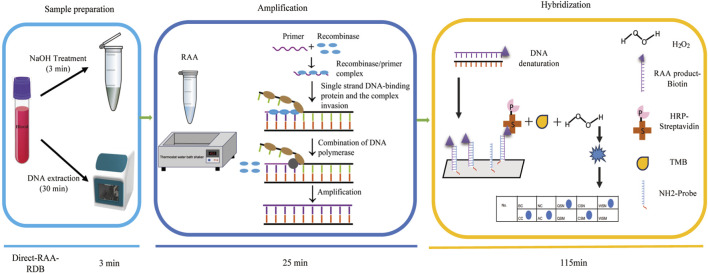
Schematic diagram of the Direct-RAA-RDB method.

Freshly obtained whole-blood samples were pretreated with 0.1 M NaOH for 3 min, and then added to the amplification reaction system (Figure 1). After incubation, 5 µL of the lysed sample was directly added to the RAA reaction mixture without neutralization step.

### 2.5 Establishment of the RAA system

RAA was performed using the Basic RAA kit (Hangzhou Zhongce Bio-Sci & Tech Co., Ltd, China). Each reaction was carried out in a total volume of 50 µL. The reaction mixture was prepared by adding 25 µL of Buffer A, forward and reverse primers (10 µM each), and ddH_2_O to the lyophilized tubes provided in the kit. Subsequently, 5 µL of amplification template and the required volume of Buffer B were added. Once the reaction solution was thoroughly mixed and centrifuged at low speed, the tubes were incubated in a constant-temperature metal bath or water bath shaker (Figure 1).

### 2.6 Gene chip preparation

To prepare the probe working solution, the synthesized probes were diluted with 0.5 mol/L sodium bicarbonate to a designated concentration for spotting. To fix the probes, nylon membranes were immersed in 1% hydrochloric acid for 5–10 min for pre-activation. The membranes were then incubated in a 10% EDAC solution (in ddH_2_O) for 30 min to activate the surface carboxyl groups, washed with pure water, and air-dried at room temperature. The activated membranes were spotted with the working solution at a volume of 0.5 μL per drop. Following the spotting process, the membranes were allowed to react at room temperature for 20–60 min. The reaction was terminated using 0.1 mol/L sodium hydroxide. The prepared gene chips were stored at 4 °C.

### 2.7 Reverse dot blot hybridization

The RAA products were denatured at 98 °C for 10 min and added to the hybridization solution (0.5×SSC and 0.1%SDS in ddH_2_O) for complementary binding to the immobilized probes on the nylon membrane chips. The biotin-labeled amplification products were incubated with HRP-streptavidin (0.25 μg/mL) for 30 min. A colorimetric reaction was initiated using hydrogen peroxide and the substrate TMB (0.1 mg/mL) for 15 min, resulting in a blue spot on the membrane strip at the corresponding probe position (Figure 1). To maximize the removal of non-specifically bound amplification products, the membrane strips were washed multiple times by washing solution (2×SSC and 0.1%SDS in ddH_2_O). The shaking speed was 50 rpm during the hybridization process.

### 2.8 System optimization and performance verification

To determine the optimal reaction conditions, various parameters were evaluated. The ratios of whole blood to 0.1 M NaOH in the sample pretreatment were tested at 1:0, 1:1, 1:2, 1:3, 1:4, and 1:5. RAA conditions were assessed at temperatures of 37, 38, 39, 40, and 41 °C, with incubation times of 20, 25, 30, 35, and 40 min. Primer volumes of 1.0, 1.5, 2.0, 2.5, and 3.0 µL, and Buffer B volumes of 1.5, 2.0, 2.5, 3.0, and 3.5 µL were also tested. For the hybridization system, probe concentrations of 2.5, 5, 7.5, 10, 20, and 30 µM were evaluated at hybridization times of 15, 30, 45, and 60 min, and temperatures of 45, 48, 50, and 52 °C. Grayscale values were calculated using ImageJ.

Performance validation included the assessment of accuracy, specificity, reproducibility, and the limit of detection. To evaluate clinical accuracy, the genotypes of clinical samples were determined and compared with results obtained from the thalassemia gene test kit and Sanger sequencing. The overall negative and positive concordance rates were calculated to evaluate the accuracy of the method.

## 3 Results

### 3.1 RAA primer design

**FIGURE 2:**
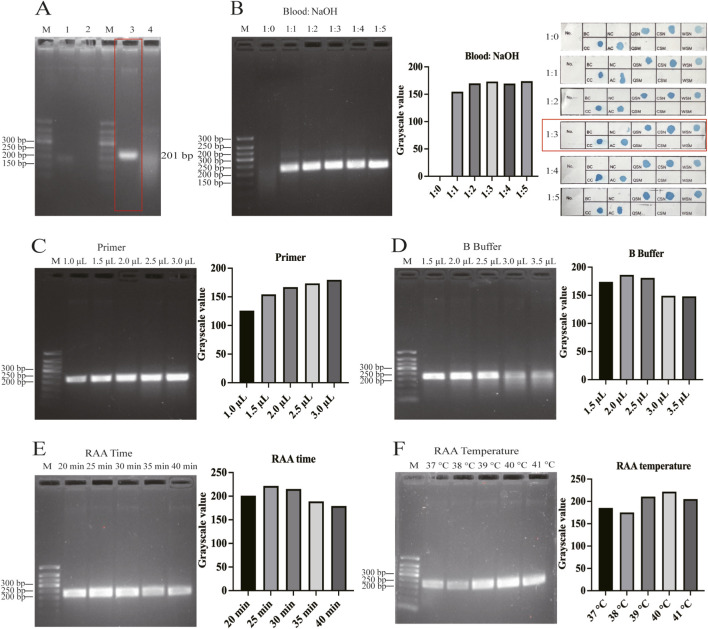
Establishment and optimization of Direct-RAA amplification conditions. **(A)** Gel electrophoresis results for Recombinase-Aided Amplification (RAA) primer screening. Lanes 1 and 2 show the results for the sample and blank control for primer pair a, respectively; lanes 3 and 4 show the results for the sample and blank control for primer pair b, respectively. Primer pair b, targeting a fragment of 201 bp, was selected. **(B)** Optimization of the ratio of whole blood to NaOH in sample pretreatment, including gel electrophoresis results, statistical analysis of electrophoresis band grayscale values, and hybridization results. **(C)** Optimization of primer volume, including gel electrophoresis results and statistical analysis of electrophoresis band grayscale values. **(D)** Optimization of ion buffer (Buffer B) volume, including gel electrophoresis results and statistical analysis of electrophoresis band grayscale values. **(E)** Optimization of RAA reaction time, including gel electrophoresis results and statistical analysis of electrophoresis band grayscale values. **(F)** Optimization of RAA reaction temperature, including gel electrophoresis results and statistical analysis of electrophoresis band grayscale values.

The optimal primer length for RAA is typically between 28 and 35 nucleotides, with a preferred amplification product size ranging from 80 to 300 bp. Following the screening of multiple primer pairs, a specific set yielding a product with a length of 201 bp was selected. This primer pair can distinguish between the α1 and α2 globin genes and encompasses the three target mutation sites: α^WS^α, α^CS^α, and α^QS^α. The primer sequences are detailed in Table 1. Agarose gel electrophoresis showed a single, distinct band, indicating that the primer pair possesses high specificity and amplification efficiency (Figure 2A).

### 3.2 Probe design and validation

Based on the α2 globin gene sequence obtained from NCBI and the specific mutations in the α^WS^α, α^CS^α, and α^QS^α variants, multiple mutant and wild-type probes were designed for each point mutation. Following the screening process, one mutant and one wild-type probe that demonstrated stable binding without non-specific hybridization were selected for each site. All selected probes produced consistent and distinct hybridization signals. The system incorporated four controls: a color system control (CC), a blank control (BC), a positive control (PC), and a negative control (NC). The CC consisted of a base sequence with one end linked to–NH_2_ and the other to biotin, and serves as a colorimetric reference for the system. The PC comprised a normal base sequence from within the amplified fragment, while the NC was a base sequence located outside the target amplification region. The probe sequences are detailed in Table 1.

### 3.3 Establishment and optimization of the RAA system

The RAA system was optimized to enhance both assay efficiency and specificity. The ratio of whole blood to 0.1 M NaOH during sample pretreatment was evaluated across six ratios: 1:0, 1:1, 1:2, 1:3, 1:4, and 1:5. Based on electrophoresis and hybridization results, ratios between 1:3 and 1:5 were found to be ideal. To account for the inherent variability among clinical samples, a ratio of 1:3 was selected as the optimal concentration (Figure 2B).

The primer volume within the RAA system was also optimized using a stock concentration of 10 µM. Different volumes—1.0, 1.5, 2.0, 2.5, and 3.0 µL—were tested. As the primer volume increased, the intensity of the target band gradually strengthened. When the volume reached 2.0 µL, the intensity of the electrophoresis band plateaued, with no significant further increases observed. Consequently, a final volume of 2 µL was selected for the primers (Figure 2C).

The volume of Buffer B (ion buffer) added to the RAA system was optimized by testing different volumes: 1.5, 2.0, 2.5, 3.0, and 3.5 µL. The intensity of the amplicon band and the corresponding grayscale values were highest when 2.0 µL of Buffer B was added. As the volume of Buffer B increased beyond this point, the intensity of the band decreased. Therefore, 2.0 µL was determined to be the optimal volume for Buffer B (Figure 2D).

The isothermal amplification duration of RAA is significantly shorter than that of conventional PCR. To optimize this parameter, various incubation times (20, 25, 30, 35, and 40 min) were evaluated. The intensity of the amplification band and the grayscale value peaked at 25 min. As the amplification time increased beyond 25 min, a decrease in band intensity was observed. Consequently, the optimized RAA reaction time was determined to be 25 min (Figure 2E).

The RAA isothermal reaction was conducted at a fixed temperature throughout the process. To optimize the amplification temperature within the RAA system, reactions were performed at 37, 38, 39, 40, and 41 °C. The intensity of the electrophoresis band and the corresponding grayscale value were highest at 40 °C. Therefore, the optimal reaction temperature was set to 40 °C (Figure 2F).

The final optimized amplification reaction conditions are summarized in Table 2.

**TABLE 2:** Direct-RAA reaction system.

RAA components		RAA reaction condition	
Component	Volume	Temperature	Time
Powder	1 aliquot	40 °C	25 min
Buffer A	25 μL		
Buffer B	2.0 μL		
10 μmol/L A2F	2.0 μL		
10 μmol/L A2R	2.0 μL		
H_2_O	14 μL		
NDA	5 μL		

### 3.4 Optimization of the reverse dot blot hybridization system

**FIGURE 3:**
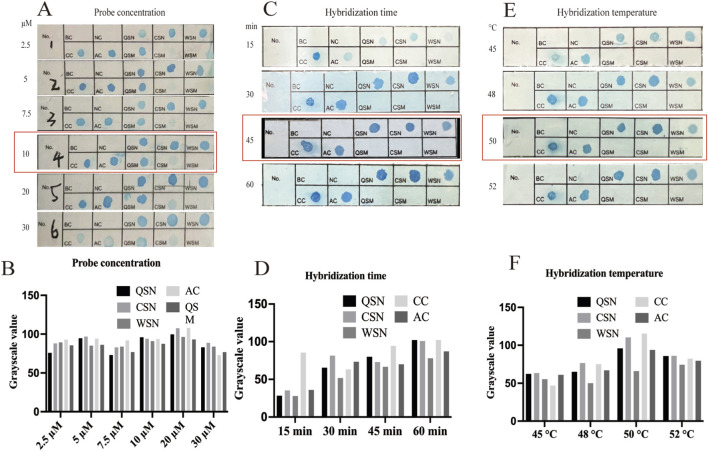
Establishment and optimization of RDB hybridization conditions. **(A)** Hybridization results for probe concentration screening on the hybridization membrane. **(B)** Statistical analysis of the grayscale values for hybridization spots in probe concentration screening. **(C)** Hybridization results for hybridization duration screening. **(D)** Statistical analysis of grayscale values for hybridization spots in hybridization duration screening. **(E)** Hybridization results for hybridization temperature screening. **(F)** Statistical analysis of grayscale values for hybridization spots in hybridization temperature screening.

The concentration of probes significantly influences the intensity of the colorimetric spots following hybridization. To determine the optimal probe concentration for the membrane, concentrations of 2.5, 5, 7.5, 10, 20, and 30 µM were evaluated. Visual results and grayscale value analysis indicated that 10 and 20 µM provided the highest signal quality. Considering both cost-efficiency and colorimetric intensity, a final concentration of 10 µM was selected (Figures 3A,B).

The hybridization duration was optimized by testing 15, 30, 45, and 60 min. As the hybridization time increased, the blue spots on the membrane gradually intensified. At 45 min, the spots demonstrated high intensity, and the grayscale values were relatively high. Therefore, a hybridization time of 45 min was chosen (Figures 3C,D).

Hybridization temperature dictates the binding specificity between complementary single-stranded DNA. To optimize this parameter, temperatures of 45, 48, 50, and 52 °C were evaluated. As the temperature increased, the blue spots became more distinct. To ensure high stringency and avoid non-specific binding, a final hybridization temperature of 50 °C was selected (Figures 3E,F).

### 3.5 Performance validation

**FIGURE 4:**
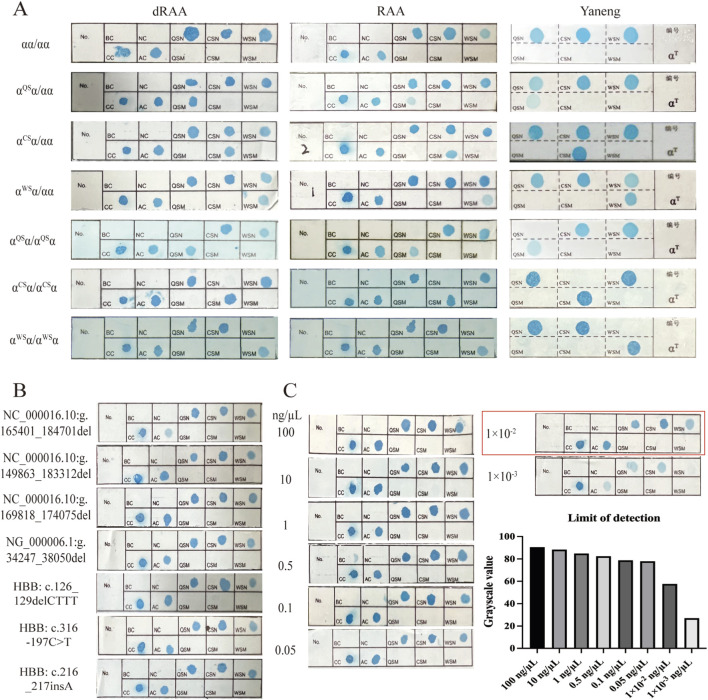
Performance evaluation of the Direct-RAA-RDB system. **(A)** Hybridization results for accuracy evaluation, comparing the results of non-deletion α-thalassemia genotyping between the Direct-RAA-RDB, RAA-RDB, and commercial PCR-RDB detection systems. **(B)** Specificity evaluation using samples with other thalassemia genotypes. **(C)** Detection limit determination, including hybridization graphs and statistical analysis of grayscale values.

To validate the newly established system, accuracy was assessed using samples containing α^WS^α, α^CS^α, and α^QS^α mutations in both heterozygous and homozygous forms, alongside wild-type samples. The Direct-RAA-RDB detection system was compared with the standard RAA-RDB system and the commercial Yaneng Bio reagent kit (PCR-RDB). The results showed complete genotype concordance among all three methods (Figure 4A). The system accurately detected the target genotypes without producing non-specific bands. The resulting signals were distinct and high in intensity, allowing for the clear differentiation between heterozygous and homozygous mutations.

To evaluate the specificity of the system, samples representing other thalassemia genotypes were selected. These included three β-thalassemia mutations (HBB: c.126_129delCTTT, HBB: c.316–197C>T, and HBB: c.216_217insA) and four deletion-type α-thalassemia mutations (NG_000006.1:g.34247_38050del, NC_000016.10:g.169818_174075del, NC_000016.10:g.165401_184701del, and NC_000016.10:g.149863_183312del). The system was tested to ensure it would not incorrectly identify these variants as non-deletion α-thalassemia. Following hybridization, no non-deletion α-thalassemia genotypes were detected, confirming the specificity of the method (Figure 4B).

To determine the limit of detection for the system, a wild-type sample with a DNA concentration of 100 ng/μL, as measured by NanoDrop, was serially diluted across eight gradients. The Direct-RAA-RDB system was employed to detect these diluted samples. The lowest concentration at which the genotype could be accurately identified was determined to be 1 × 10^−2^ ng/μL (Figure 4C).

**FIGURE 5:**
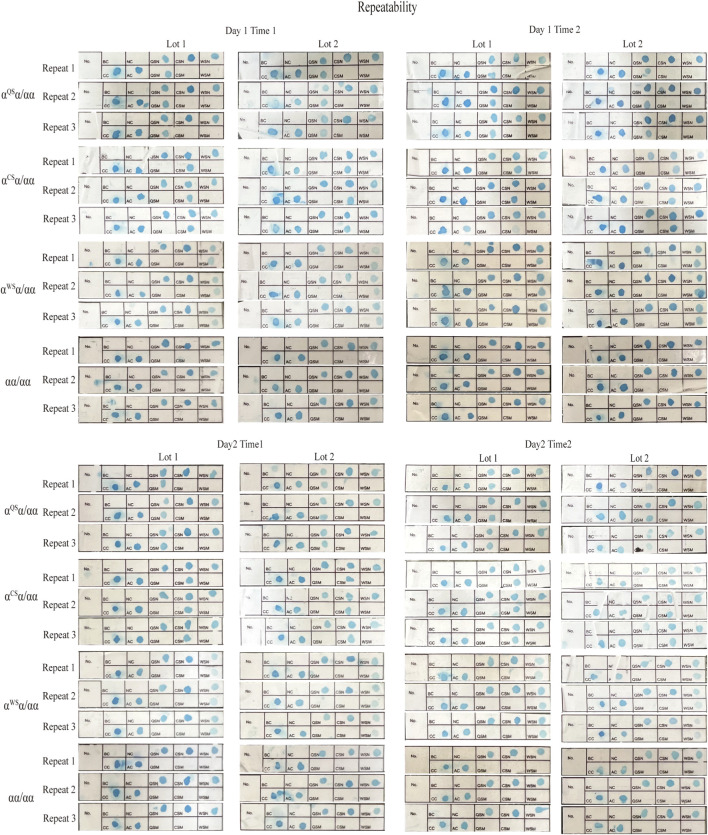
Repeatability testing for the Direct-RAA-RDB system in non-deletion α-thalassemia.

To validate the reproducibility of the system, wild-type samples and heterozygous samples for α^WS^α, α^CS^α, and α^QS^α were evaluated. Two batches of membranes were compared, with each sample tested in triplicate. The experiment was repeated twice daily over two consecutive days. The results showed that the Direct-RAA-RDB system provided stable and accurate genotype detection across all replicates and batches (Figure 5).

### 3.6 Clinical evaluation

**FIGURE 6:**
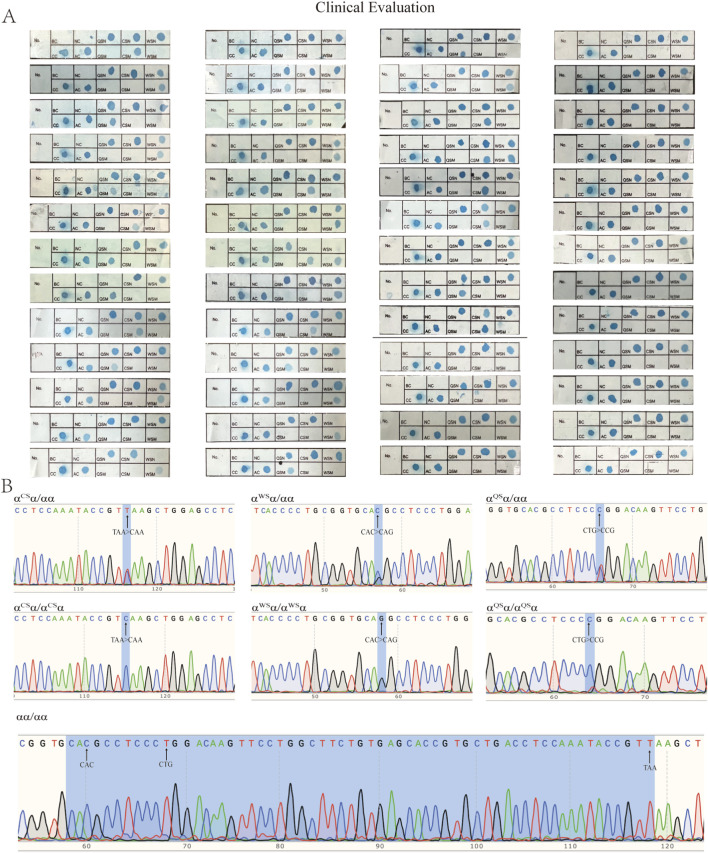
Clinical evaluation of the Direct-RAA-RDB system of non-deletion α-thalassemia. **(A)** Hybridization results for 52 clinical samples, including 35 positive and 17 negative samples. **(B)** Sequencing results for a subset of the samples.

To evaluate the clinical performance of the Direct-RAA-RDB system, 35 positive samples and 17 negative samples with confirmed genotypes were analyzed (Figure 6A). The assessment yielded a 100% positive concordance rate, a 100% negative concordance rate, and an overall concordance rate of 100%. The calculated Kappa value was 1, indicating almost perfect agreement (Table 3). Additionally, a subset of samples was subjected to Sanger sequencing. The genotypes obtained through sequencing matched those detected by the Direct-RAA-RDB system (Figure 6B).

**TABLE 3:** Comparison of the results of the Direct-RAA-RDB and PCR-RDB assays.

Methods		Direct-RAA-RDB			Positive (%)	Negative (%)	Kappa
		Positive	Negative	Total			
PCR-RDB	Positive	35	0	35	100	100	1
	Negative	0	17	17			
	Total	35	17	52			

## 4 Discussion

In this study, we successfully established a Direct-RAA-RDB platform for the simultaneous detection of three common non-deletion α-thalassemia point mutations (α^WS^α, α^CS^α, α^QS^α) directly from whole blood, eliminating the need for DNA extraction, building on our team’s previous application of this platform to β-thalassemia. Our results demonstrate that this platform achieves 100% concordance with commercial PCR-RDB kits and Sanger sequencing, exhibits high sensitivity (0.01 ng/μL), and significantly reduces the total assay time (143 min vs. 310 min for PCR-RDB) (Table 4). These findings address the critical need for a rapid, accurate, and equipment-light method suitable for POCT and resource-limited settings.

**TABLE 4:** Comparison of Testing Timeliness between the PCR-RDB and Direct-RAA-RDB methods.

Method	DNA extraction (min)	Amplification (min)	Denaturation (min)	RDB (min)	Total time (min)
Traditional PCR-RDB	30	150	10	120	310
Direct-RAA-RDB	3	25	10	105	143

A critical innovation of our system is the use of a brief (3 min), room-temperature NaOH pretreatment to directly prepare whole blood for RAA amplification, bypassing conventional DNA purification. We systematically optimized the blood-to-NaOH ratio and found that a 1:3 ratio maximized inhibitor inactivation without compromising amplification efficiency. This finding aligns with reports that NaOH effectively inactivates PCR inhibitors such as hemoglobin and lactoferrin (Liu et al., 2018). Importantly, compared to traditional purification methods, our approach reduces sample preparation time (Connelly et al., 2014) by approximately 20 min per batch and minimizes cross-contamination risks by eliminating multiple washing and elution steps. This streamlined workflow, compatible with 96-well plate formats, also enables high-throughput applications without the need for specialized nucleic acid extraction instruments.

RAA is analogous to RPA in principle and enzyme composition, differing primarily in recombinase source (bacterial/fungal vs. T4 phage). Although the optimal pH for RAA has not been directly reported, RPA functions optimally at pH 7.5–8.0 (Kojima et al., 2021). Consistent with other isothermal systems (e.g., alkaline SIL-B in SmartAmp2) (Victor et al., 2009),our Direct-RAA-RDB directly adds alkaline-pretreated samples without neutralization. The RAA buffer (Tris-HCl) provides sufficient buffering capacity, as the alkaline lysate comprises only 2.5% of the total volume, maintaining optimal pH for enzymatic activity. Thus, omitting neutralization simplifies the workflow without compromising amplification efficiency. Notably, a counterintuitive decrease in RAA product intensity was observed after 25 min of incubation. First, prolonged amplification promotes primer-dimer and off-target product formation, which compete with the target amplicon for primers, polymerase, and SSB proteins (Piepenburg et al., 2006). Second, accumulated inorganic pyrophosphate chelates Mg^2+^, an essential polymerase cofactor, thereby reducing amplification efficiency (Wang et al., 2022). Third, nonspecific by-products generated during extended reactions compete for limited reaction resources (Munawar et al., 2020). Importantly, the 3-min NaOH pretreatment (0.1 M, pH ∼13) effectively inactivates endogenous nucleases via irreversible protein denaturation, as evidenced by sharp, intact bands at 20–25 min without smearing. Thus, the decline after 25 min is attributable to reaction component depletion and byproduct accumulation rather than nuclease-driven degradation.

Compared to other isothermal amplification techniques, RAA offers distinct practical advantages (Lin et al., 2022). While LAMP typically requires 60–90 min for amplification, our RAA system completes this step in just 25 min at 40 °C. Furthermore, unlike qPCR-based methods that are limited by fluorescence channel numbers and require expensive thermal cyclers, our RDB-based readout enables simultaneous detection of multiple targets in a single hybridization reaction using only a constant-temperature water bath shaker (Saiki et al., 1989; Y et al., 2019). This feature is particularly advantageous for thalassemia genotyping, where multiple point mutations must be distinguished. Our data show that the Direct-RAA-RDB system not only matches the accuracy of PCR-RDB (Kappa = 1) but also provides visual, intuitive results akin to lateral flow assays, making it well-suited for laboratories with limited medical equipment (Ghosh et al., 2022).

Clinical validation using 52 blinded samples (35 positives, 17 negatives) yielded 100% concordance rates, and a Kappa value of 1, indicating perfect agreement with the reference method. All samples were randomly selected from a pre-genotyped biobank at the Guangxi Zhuang Autonomous Region People’s Hospital and were previously characterized by PCR-RDB. Regarding statistical power, despite the limited sample size, the 95% confidence intervals ranged from 89.9% to 100% for sensitivity, 80.8%–100% for specificity, and 93.1%–100% for overall agreement (Clopper-Pearson method). All lower bounds exceeded 80% for all performance metrics. Furthermore, all samples were sourced from Guangxi, a multi-ethnic region encompassing Han, Zhuang, Yao, Miao, and Dong populations; therefore, the cohort inherently reflects regional ethnic diversity, though multi-center prospective studies with larger cohorts are needed to further confirm diagnostic accuracy and generalizability.

Several limitations should be acknowledged. First, while the NaOH pretreatment inactivates major inhibitors, its efficiency may vary with sample quality (e.g., lipemic or icteric specimens), which was not systematically evaluated. Second, the open-well format poses a cross-contamination risk for highly sensitive isothermal amplifications, necessitating future optimization of reaction vessels or closed-system designs. Third, although validated for whole blood, the platform’s applicability to other sample types (dried blood spots, amniotic fluid, saliva) requires separate validation. Fourth, the current panel covers only three non-deletion α-thalassemia mutations; other clinically relevant variants (rare point mutations, large deletions) are not detected. Thus, a negative result cannot exclude other thalassemia mutations. Future work will focus on expanding mutation coverage and adapting the platform for multiplexed pathogen detection. Despite these limitations, the Direct-RAA-RDB platform represents a rapid, cost-effective (Table 5), and accessible screening tool for non-deletion α-thalassemia in resource-limited settings.

**TABLE 5:** Comparison of cost between PCR-RDB and Direct-RAA-RDB.

Method	Reagent cost (1 sample)			Equipment cost			Labor time/Cost
	Nucleic acid extraction	Nucleic acid amplification	Hybridization	Nucleic acid extraction instrument	PCR instrument	Hybridization instrument	
Traditional PCR-RDB	$1–2	$2	$1.5	$2,857-$5,714	$1,143-$2,286	$1,143-$2,286	120 min/$30
Direct-RAA-RDB	$0.5	$2.5	$1.5	$0	$68-$100		60 min/$15

Reagent costs were estimated based on commercial suppliers' list prices: the PCR-RDB, kit from Yaneng Bio, the nucleic acid extraction kit from TIANGEN, and the RAA kit from Zhongce Bio. equipment costs were calculated as the purchase price per instrument, with prices for the nucleic acid extractor and PCR, thermal cycler referenced from Tianlong Technology Co., Ltd., and the hybridization instrument referenced from Yaneng Bio. Labor time was defined as the hands-on time required during the experimental procedure. Labor cost was calculated as the hands-on time per batch multiplied by the hourly wage of a laboratory technician ($15/h). “$0” indicates that the instrument is not required. All prices may vary depending on region and time period.

## 5 Conclusion

In conclusion, we have established a gene detection platform, designated as Direct-RAA-RDB, that eliminates the requirement for DNA extraction by using whole blood as a direct template. This platform integrates RAA with RDB technology to provide a rapid, highly sensitive, convenient, and high-throughput method for genetic analysis. We have successfully applied this platform to the detection of three non-deletion α-thalassemia mutations: α^WS^α, α^CS^α, and α^QS^α. Through both performance and clinical evaluations, the system demonstrated excellent accuracy and practical applicability. The platform is not only suitable for large-scale experiments but is particularly well-suited for use in grassroots laboratories and POC settings where medical equipment and experimental conditions may be limited. This platform should be applicable to a wider range of sample types, genetic targets.

## Data Availability

The original contributions presented in the study are included in the article/supplementary material, further inquiries can be directed to the corresponding authors.

## References

[B1] Connelly C. M. Porter L. R. TerMaat J. R. (2014). PCR amplification of a triple-repeat genetic target directly from whole blood in 15 minutes as a proof-of-principle PCR study for direct sample analysis for a clinically relevant target. BMC Med. Genet. 15, 130. 10.1186/s12881-014-0130-5 25495904 PMC4411754

[B2] Custer G. F. Dibner R. R. (2020). Modified methods for loading of high-throughput DNA extraction plates reduce potential for contamination. J. Vis. Exp. 160, e61405. 10.3791/61405 32568245

[B3] Fan X. Li L. Zhao Y. Liu Y. Liu C. Wang Q. (2020). Clinical validation of two recombinase-based isothermal amplification assays (RPA/RAA) for the rapid detection of African swine fever virus. Front. Microbiol. 11, 1696. 10.3389/fmicb.2020.01696 32793160 PMC7385304

[B4] Gao J. Huang S. Jiang J. Miao Q. Zheng R. Kang Y. (2025). Dual-CRISPR/Cas12a-assisted RT-RAA visualization system for rapid on-site detection of nervous necrosis virus (NNV). Anal. Chim. Acta. 1335, 343469. 10.1016/j.aca.2024.343469 39643320

[B5] Ghosh P. Chowdhury R. Hossain M. E. Hossain F. Miah M. Rashid M. U. (2022). Evaluation of recombinase-based isothermal amplification assays for point-of-need detection of SARS-CoV-2 in resource-limited settings. Int. J. Infect. Dis. 114, 105–111. 10.1016/j.ijid.2021.11.007 34758392 PMC8572376

[B6] Guo Z. Li K. Qiao S. Chen X. X. Deng R. Zhang G. (2020). Development and evaluation of duplex TaqMan real-time PCR assay for detection and differentiation of wide-type and MGF505-2R gene-deleted African swine fever viruses. BMC Vet. Res. 16 (1), 428. 10.1186/s12917-020-02639-2 33167979 PMC7654620

[B7] Guo Z. Xing G. Li P. Jin Q. Lu Q. Zhang G. (2023). Development and application of a recombinase-aided amplification and lateral flow assay for rapid detection of pseudorabies virus from clinical crude samples. Int. J. Biol. Macromol. 224, 646–652. 10.1016/j.ijbiomac.2022.10.153 36283557

[B8] Hu T. Ke X. Li W. Lin Y. Liang A. Ou Y. (2023). CRISPR/Cas12a-Enabled multiplex biosensing strategy Via an affordable and visual nylon membrane readout. Adv. Sci. (Weinh) 10 (2), e2204689. 10.1002/advs.202204689 36442853 PMC9839848

[B9] Kojima K. Juma K. M. Akagi S. Hayashi K. Takita T. O'Sullivan C. K. (2021). Solvent engineering studies on recombinase polymerase amplification. J. Biosci. Bioeng. 131 (2), 219–224. 10.1016/j.jbiosc.2020.10.001 33177003

[B10] Li J. Cui H. Zhang Y. Wang X. Liu H. Mu Y. (2024). A rapid detection method for H3 avian influenza viruses based on RT-RAA. Anim. (Basel) 14 (17), 2601. 10.3390/ani14172601 39272386 PMC11393923

[B11] Lin H. Liang Y. Zou L. Li B. Zhao J. Wang H. (2022). Combination of isothermal recombinase-aided amplification and CRISPR-Cas12a-Mediated assay for rapid detection of major severe acute respiratory syndrome coronavirus 2 variants of concern. Front. Microbiol. 13, 945133. 10.3389/fmicb.2022.945133 35836420 PMC9274097

[B12] Liu X. Zhang C. Zhao M. Liu K. Li H. Li N. (2018). A direct isothermal amplification system adapted for rapid SNP genotyping of multifarious sample types. Biosens. Bioelectron. 115, 70–76. 10.1016/j.bios.2018.05.021 29803102 PMC7126597

[B13] Munawar M. A. Martin F. Toljamo A. Kokko H. Oksanen E. (2020). RPA-PCR couple: an approach to expedite plant diagnostics and overcome PCR inhibitors. Biotechniques 69 (4), 270–280. 10.2144/btn-2020-0065 32815734

[B14] Piepenburg O. Williams C. H. Stemple D. L. Armes N. A. (2006). DNA detection using recombination proteins. PLoS Biol. 4 (7), e204. 10.1371/journal.pbio.0040204 16756388 PMC1475771

[B15] Saiki R. K. Walsh P. S. Levenson C. H. Erlich H. A. (1989). Genetic analysis of amplified DNA with immobilized sequence-specific oligonucleotide probes. Proc. Natl. Acad. Sci. U. S. A. 86 (16), 6230–6234. 10.1073/pnas.86.16.6230 2762325 PMC297811

[B16] Sun Y. Tang D. Li N. Wang Y. Yang M. Shen C. (2024). Development of a rapid epstein-Barr virus detection System based on recombinase polymerase amplification and a lateral flow assay. Viruses 16 (1), 106. 10.3390/v16010106 38257806 PMC10818573

[B17] Victor S. T. Lezhava A. Ishidao T. Endo R. Mitani Y. Kawaoka Y. (2009). Isothermal single nucleotide polymorphism genotyping and direct PCR from whole blood using a novel whole-blood lysis buffer. Mol. Diagn Ther. 13 (6), 383–387. 10.1007/BF03256344 19925036

[B18] Wang J. Konigsberg W. H. (2022). Two-Metal-Ion catalysis: inhibition of DNA polymerase activity by a third Divalent metal ion. Front. Mol. Biosci. 9, 824794. 10.3389/fmolb.2022.824794 35300112 PMC8921852

[B19] Y K. Id O. P S. Id O. X M. Id- Orcid X. (2019). PCR-reverse dot blot human papillomavirus genotyping as a primary screening test. J. Gynecol. Oncol. 30 (3), e29. 10.3802/jgo.2019.30.e29 30887754 PMC6424850

[B20] Ye C. Zhou X. Wei Y. Zhao Y. Xie M. Liu X. (2025). A direct multiplex isothermal amplification-reverse dot blot hybridization system for beta-thalassemia diagnosis. Ann. Hematol. 104 (12), 6147–6159. 10.1007/s00277-025-06711-5 41249661 PMC12764633

[B21] Zhang Q. Xiao H. Yan L. (2021). PCR-reverse blot hybridization assay in respiratory specimens for rapid detection and differentiation of mycobacteria in HIV-negative population. BMC Infect. Dis. 21 (1), 264. 10.1186/s12879-021-05934-x 33726688 PMC7962079

[B22] Zhao S. Zhang Q. Wang X. Li W. Juma S. Berquist R. (2023). Development and performance of recombinase-aided amplification (RAA) assay for detecting Schistosoma haematobium DNA in urine samples. Heliyon 9 (12), e23031. 10.1016/j.heliyon.2023.e23031 38144328 PMC10746445

